# Supplementary material for: A fault-intrusion-tolerant system and deadline-aware algorithm for scheduling scientific workflow in the cloud
Source: PeerJ Comput Sci. 2021 Nov 2;7:e747. doi: 10.7717/peerj-cs.747 (PMC8576554; doi:10.7717/peerj-cs.747)
Supplement: Supplemental Information 6 [file peerj-cs-07-747-s006.zip › Myproject1.1/docs/org/cloudbus/cloudsim/UtilizationModel.html]

UtilizationModel


JavaScript is disabled on your browser.


- Overview
- Package
- Class
- Use
- Tree
- Deprecated
- Index
- Help

- Prev Class
- Next Class

- Frames
- No Frames

- All Classes

- Summary:
- Nested |
- Field |
- Constr |
- Method

- Detail:
- Field |
- Constr |
- Method


org.cloudbus.cloudsim

## Interface UtilizationModel

- All Known Implementing Classes:
  :   UtilizationModelFull, UtilizationModelNull, UtilizationModelPlanetLabInMemory, UtilizationModelStochastic

  ---

    

  ```
  public interface UtilizationModel
  ```

  The UtilizationModel interface needs to be implemented in order to provide a fine-grained control
  over resource usage by a Cloudlet.

  Since:
  :   CloudSim Toolkit 2.0

- - ### Method Summary

    Methods

    | Modifier and Type | Method and Description |
    | --- | --- |
    | `double` | `getUtilization(double time)` Returns utilization in percents according to the time. |

- - ### Method Detail


    - #### getUtilization

      ```
      double getUtilization(double time)
      ```

      Returns utilization in percents according to the time.

      Parameters:
      :   `time` - the time

      Returns:
      :   utilization percentage


- Overview
- Package
- Class
- Use
- Tree
- Deprecated
- Index
- Help

- Prev Class
- Next Class

- Frames
- No Frames

- All Classes

- Summary:
- Nested |
- Field |
- Constr |
- Method

- Detail:
- Field |
- Constr |
- Method
